# Cationic Liposomes Formulated with Synthetic Mycobacterial Cordfactor (CAF01): A Versatile Adjuvant for Vaccines with Different Immunological Requirements

**DOI:** 10.1371/journal.pone.0003116

**Published:** 2008-09-08

**Authors:** Else Marie Agger, Ida Rosenkrands, Jon Hansen, Karima Brahimi, Brian S. Vandahl, Claus Aagaard, Kerstin Werninghaus, Carsten Kirschning, Roland Lang, Dennis Christensen, Michael Theisen, Frank Follmann, Peter Andersen

**Affiliations:** 1 Adjuvant Research, Department of Infectious Disease Immunology, Statens Serum Institut, Copenhagen, Denmark; 2 Institute of Medical Microbiology, Immunology and Hygiene, Technical University Munich, Munich, Germany; Federal University of São Paulo, Brazil

## Abstract

**Background:**

It is now emerging that for vaccines against a range of diseases including influenza, malaria and HIV, the induction of a humoral response is insufficient and a substantial complementary cell-mediated immune response is necessary for adequate protection. Furthermore, for some diseases such as tuberculosis, a cellular response seems to be the sole effector mechanism required for protection. The development of new adjuvants capable of inducing highly complex immune responses with strong antigen-specific T-cell responses in addition to antibodies is therefore urgently needed.

**Methods and Findings:**

Herein, we describe a cationic adjuvant formulation (CAF01) consisting of DDA as a delivery vehicle and synthetic mycobacterial cordfactor as immunomodulator. CAF01 primes strong and complex immune responses and using ovalbumin as a model vaccine antigen in mice, antigen specific cell-mediated- and humoral responses were obtained at a level clearly above a range of currently used adjuvants (Aluminium, monophosphoryl lipid A, CFA/IFA, Montanide). This response occurs through Toll-like receptor 2, 3, 4 and 7-independent pathways whereas the response is partly reduced in MyD88-deficient mice. In three animal models of diseases with markedly different immunological requirement; *Mycobacterium tuberculosis* (cell-mediated), *Chlamydia trachomatis* (cell-mediated/humoral) and malaria (humoral) immunization with CAF01-based vaccines elicited significant protective immunity against challenge.

**Conclusion:**

CAF01 is potentially a suitable adjuvant for a wide range of diseases including targets requiring both CMI and humoral immune responses for protection.

## Inrtoduction

Vaccination is one of the most successful and cost-effective public health interventions with more than 2 million deaths being prevented due to immunization each year [1]. Today, world-wide campaigns include vaccinations against polio, diphtheria, pertussis and tetanus and in the last two decades new vaccines have reached the global market e.g. the vaccine against *Haemophilus influenzae* type b. However, a number of widespread and serious diseases have so far escaped vaccination attempts. International health priorities such as HIV, TB and malaria are included among the targets but also the threat of new emerging flu pandemics has received global awareness and is a highly active area of vaccine research.

Generally, the main focus for vaccine research has been antigen discovery whereas the method for inducing immune responses against these antigens has received relative limited attention. Therefore, today one of the major obstacles in developing next generation vaccines is the need for effective adjuvants available for clinical trials. The paucity of adjuvants is reflected by the fact that aluminium compounds identified as having immunostimulatory properties more than 70 years ago remain the only type of adjuvant licensed for world-wide usage. In addition, the oil-in-water formulation designated MF59 has received licensure in some countries as part of an influenza vaccine along with virosomes used in both influenza and hepatitis A vaccines [2], [3]. However, both of these adjuvants are characterised by inducing humoral immune responses and are thus effective in elevating serum antibody titers whereas their ability to elicit cell-mediated immune (CMI) responses is limited. As many of the remaining difficult disease targets rely on varying levels of CMI responses with or without an associated humoral response there is a large unmet need for novel CMI inducing adjuvants. TB and HIV both belong to this category of global health problems that are crucially dependent on a strong CMI response for protection but also many of the existing vaccines may benefit from an improved adjuvant technology that would stimulate both arms of the immune system. This is illustrated by influenza where antibodies neutralize the infectivity of the virus and the cytotoxic T-cells reduce viral spread and thereby serve to enhance the recovery from influenza [4].

Herein, we evaluated the immunogenicity and efficacy of a newly developed liposomal adjuvant, designated cationic adjuvant formulation (CAF01) [5]. This adjuvant is based on liposomes formed by N,N′-dimethyl-N,N′-dioctadecylammonium (DDA) with the synthetic mycobacterial immunomodulator α,α′-trehalose 6,6′-dibeheneate (TDB) inserted into the lipid bilayers. We demonstrate that compared to a panel of commercially available adjuvants, CAF01 was particular effective in generating strong cellular immune responses and in addition hereto a strong antibody response with high titers of IgG2. Experiments using TLR2, 3, 4, 7 gene-deficient mice exhibited no defects in this response whereas a reduction was observed in MyD88 knock-out mice. In three animal disease models with markedly different immunological requirement; *Mycobacterium tuberculosis* (CMI), *Chlamydia trachomatis* (CMI/humoral) and blood-stage malaria (humoral), immunization with selected candidate vaccine antigens administered in CAF01 gave rise to significant levels of protection.

## Materials and Methods

### Animals

Six- to 10-week-old female BABL/c, C57BL/6 mice were purchased from Harlan Scandinavia. Breeding pairs for mice deficient in MyD88 and TLR 3, 4 and 7 were kindly provided by Dr. S. Akira (Osaka, Japan). TLR2, TLR3, TLR4 and TLR7 knockout mice were backcrossed onto a C57BL/6 background to obtain TLR2, TLR3, TLR4 and TLR7 quadruple-deficient mice. All mice were backcrossed for at least six generations to C57BL/6, before TLR-deficient mice were successively interbred to generate mice homozygous for the knockout allele in the loci for TLR2, TLR3, TLR4 and TLR7. Mice receiving a mycobacterial challenge were housed in a BSL-3 facility. All experiments were conducted in accordance with the regulations set forward by the Danish Ministry of Justice and Animal Protection Committees and in compliance with EC Directive 86/609.

### Reagents

Aluminium hydroxide (Al(OH)_3_) (2% alhydrogel) was from Brenntag Biosector (Frederikssund, Denmark), monophosphoryl lipid A from Avanti Polar Lipids (Alabaster, AL), Montanide ISA720 from Seppic (France), and Complete Freunds Adjuvant (CFA) and Incomplete Freunds Adjuvant (IFA) from Statens Serum Institut (Copenhagen, Denmark). Dimethyldioctadecylammonium (DDA) bromide and a,a′-trehalose 6,6′-dibehenate (TDB) were purchased from Avanti Polar Lipids and a stable formulation designated CAF01 prepared by the lipid film hydration method as previously described [5].

### Antigens

Ovalbumin (OVA) was obtained from Sigma. OVA CD4 T cell epitope (ISQAVHAAHAEINEAGR) and CD8 T cell epitope (SIINFEKL) were purchased through Schafer-N (Copenhagen, Denmark). The fusion protein of Ag85B and ESAT-6 was produced as a recombinant protein as previously described [6] and the major outer membrane protein (MOMP) from *Chlamydia muridarum* was expressed in the pDest17 system (Gateway, Invitrogen) and purified as described [7]. The 19kDa C-terminal protective fragment of MSP1 [8] was amplified from *Plasmodium yoelii* genomic DNA using the PYMSP1_fw (5′-CAC CGG CAC ATA GCC TCA ATA GCT TTA AAC A) and PYMSP1_rev (5′-CTA GCT GGA AGA ACT ACA GAA TAC ACC TT) primers. Amplification was carried out for 25 cycles with denaturation at 94°C for 30 sec, annealing at 55°C for 30 sec, and extension at 72°C for 2 min, using Phusion polymerase (Finnzymes, Espoo, Finland). The resulting DNA fragment was cloned into pENTR/D-TOPO (Invitrogen, Copenhagen) and subsequently into pDEST17 expression vector. The corresponding recombinant protein was purified by metal chelate affinity chromatography essentially as described [9].

### Immunization

**Figure 1:**
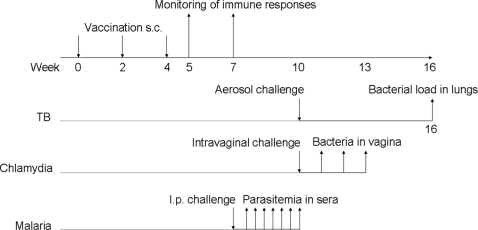
Time schedule for the immunization and infection regimen for TB, chlamydia and malaria.

Mice were immunized three times at 2-week intervals subcutaneously (s.c.) at the base of the tail using a volume of 200 µl. Alternatively, mice were injected twice at 2-week intervals in the footpad with a volume of 50 µl. All mice received a dose of 250 µg DDA/50 µg TDB or 500 µg of Al(OH)_3_ with either 100 µg of OVA, 2 µg of Ag85B-ESAT-6, 5 µg of MOMP or 10 µg of MSP1-19. OVA was also administered in combination with 250 µg of DDA, 70 µl Montanide/dose, or CFA followed by two boosts with IFA. A homogenous preparation of Montanide/OVA was prepared by using a micro-emulsifying needle (Fischer Scientific, US) [10]. DDA liposomes were prepared as previously described [11]. The experimental protocol for the three disease model has been depicted in Fig. 1.

### Detection of vaccine-specific antibodies by ELISA

Micro titers plates (Nunc Maxisorp, Roskilde, Denmark) were coated with OVA (2 µg/ml), Ag85B-ESAT-6, MOMP, or MSP1-19 (all 0.5 µg/well) in PBS overnight at 4°C. Free binding sites were blocked with 2% skim milk in PBS. Individual mouse serum from three to six mice per group was analysed in duplicate in fivefold dilutions at least 8 times in PBS containing bovine serum albumin starting with a 20-fold dilution. Horseradish peroxidase (HRP)-conjugated secondary antibodies (rabbit anti-mouse immunoglobulin G1; IgG1 and IgG2a/b/c; Zymed) diluted 1/2000 in PBS with 1% bovine serum albumin was added. After 1 h of incubation, antigen-specific antibodies were detected by TMB substrate as described by the manufacturer (Kem-En-Tec, Copenhagen, Denmark). The absorbance values were plotted as a function of the reciprocal dilution of serum samples. Reciprocal plasma dilutions corresponding to 50% maximal binding (i.e EC50) were computed using the Prism software (v. 4.00; GraphPad Software Inc.). In BALB/c mice, the IgG2a isotypes was measured whereas IgG2b and c levels were analyzed in C57BL/6 mice as the gene for IgG2a is deleted in this strain [12].

### Lymphocyte cultures

Cultures of inguinal lymph nodes or spleens were obtained by homogenizing the organ into complete RPMI (RPMI 1640 supplemented with 5×10-5 M 2-mercapthoethanol, 1 mM glutamine, 1% penicillin-streptomycin, 1% HEPES and 10% fetal calf serum all from Gibco (Invitrogen, Carlsbad, CA) and subsequently washed twice and adjusted to a final concentration of 2×10^5^ cells/well in a total volume of 200 µl/well. Antigens were used at different concentrations (ranging from 0.05 to 10 µg/ml), whereas Con A was used at a concentration of 1 µg/ml as a positive control for cell viability. Culture supernatants were harvested from parallel cultures after 72 h of incubation for the investigation of IFN-γ by ELISA performed as previously described [13].

### IFN-γ ELISPOT

96-well PVDF filter microtiters plates (MAHA S45 10 Millipore Corp, MA) were coated over-night with 4 µg/ml IFN-γ capture Ab (Pharmingen, San Diego, CA) in PBS. Plates were washed five times and blocked with RPMI medium containing 10 % FCS. Splenocytes from individual mice were added in duplicates and incubated for 48 h at 37°C, 5% CO_2_ with 5 µg of OVA. Plates were subsequently washed with 0.05% Tween in PBS and incubated with 1.25 µg/ml of anti-IFN-γ-biotin secondary Ab diluted in PBS (Clone XMG1.2; BD Pharmingen) for 2 h and phopsphatase-conjugated streptavidin (Jackson Immunoresearch Laboratories) for 45 min. The enzyme reaction was developed with 5-bromo-4-chloro-3indolylphosphate (Sigma Aldrich). Spots were counted using an automated ELISPOT Reader System and software (AID, Strassberg, Germany).

### Mycobacterial challenge

*Mycobacterium tuberculosis* Erdman were grown at 37 C in Sauton medium enriched with 0.5% glucose, 0.5 sodium pyruvate and 0.05% Tween 80. Ten weeks after the first immunization, mice were infected by the aerosol route using a Glas-Col Inhalation Exposure System (Glas-Col, Terre Haute, IN) calibrated to deliver approximately 25 colony-forming units (CFU) of *M. tuberculosis* into the airways. At different time points after infection, mice were sacrificed and organs homogenized in PBS for bacterial enumeration. Individual organs were plated in serial dilutions onto Middlebrook 7H11 agar supplemented with 2 µg of 2-thiophenecarboxylic acid hydrazide per ml to selectively inhibits the growth of BCG. The plates were incubated for 2–3 weeks at 37°C after which the number of CFUs were counted.

### Malaria challenge

*Plasmodium yoelii* 17XNL clone 1.1 was obtained from Pierre Druilhe, Institut Pasteur France and propagated in BALB/c mice. Parasitized red blood cells were kept at −80°C until use. Challenge infections were performed by intra-peritoneal injection of 10^5^ parasitized red blood cells. The course of the infection was monitored by microscopic examination of thin blood smears. *P. yoelii* infected cells were washed three times with PBS before making the smears.

### Chlamydia challenge

The *C. muridarum* strain MoPn/NiggII was purchased from the American Type Culture Collection (ATCC, Manassas, VA, USA) and propagated in HeLa-229 cells as described [14]. Chlamydia elementary bodies were harvested, purified and quantified [15] and stored at −80°C in a sucrose-phosphate-glutamate (SPG) buffer. Female C57BL/6 mice were used for vaccine experiments. One week before *C. muridarum* challenge, the mice oestrus cycle was synchronized by subcutaneous injection of 2.5 mg Medroxyprogesteronacetat (Depo-Provera; Pfizer, Ballerup, Denmark). Six weeks after the final vaccination, the mice were challenged intra-vaginally with 1.5×10^5^ inclusion forming units (IFU) (determined as 100×ID_50_) of *C. muridarum* in 10 µl SPG buffer. Vaginal swabs were obtained at 3, 7, 10, 14 and 21 days after infection. Swabs were vortexed with glass-beads in 1 ml SPG buffer and stored at −80°C until analysis. Infectious load was assessed by infection of McCoy cell monolayers with a titrated volume of the swap suspension essentially as described previously (16). Fourty hours after infection, McCoy cells were fixed and chlamydia inclusions stained with a polyclonal rabbit anti-MOMP serum, followed by a FITC conjugated swine anti-rabbit Ig (DAKO). Background staining was done with propidium iodide (Invitrogen). Swap titrations were done in duplicate. Inclusions were enumerated by visual inspection using a fluorescence microscopy.

### Statistical analyses

For analyzes of immune responses and vaccine efficacy, data were tested by analyzes of variance (more than two groups) or a t-test (two groups). When significant differences were indicated, differences between means were determined by Dunnett's multiple comparison test.

## Results

### Vaccination with CAF01 gives rise to the induction of both cell-mediated and humoral immune responses

**Figure 2:**
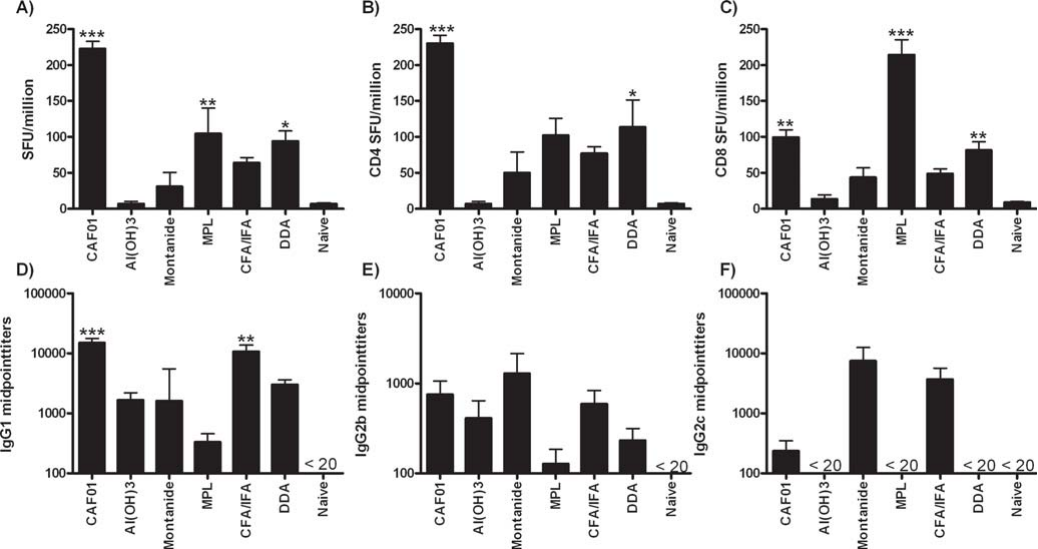
Comparison of CAF01 with other adjuvants. C57BL/6 mice (n = 4) were immunized three times with 100 µg of OVA in CAF01, Al(OH)3, Montanide, MPL, CFA/2×IFA boosting with a two-week interval. A) Three weeks after the last immunization, the number of OVA-specific cells was assessed by IFN-γ ELISPOT. Mean spot-forming units (SFU) upon stimulation with 5 µg of OVA per million cells ± SEM for each group is shown. B) SFU per million cells ± SEM upon stimulation with 5 µg of OVA CD4 T cell epitope. C) SFU per million cells ± SEM upon stimulation with 5 µg of OVA CD4 T cell epitope. D) Sera were analysed for the presence of OVA-specific IgG1, E) IgG2b, F) IgG2c antibodies by ELISA. Mean EC50 ± SEM is shown. Values marked with an asterisk are significantly different (*, *p*<0.05; **, *p*<0.01, *p*<0.001) compared to naïve controls as assessed by ANOVA and Dunnett's multiple comparison test.

The ability of CAF01 to induce both humoral and cell- mediated immune responses was analyzed using a model antigen OVA and the responses compared to other adjuvants; Montanide, MPL, DDA, Freunds complete/incomplete (CFA/IFA) adjuvant and including aluminium hydroxide (Al(OH)_3_) which is licensed for human use. C57BL/6 mice were immunized three times with 100 µg of OVA administered in adjuvant and antigen-specific antibody responses as well as T-cell responses was measured by ELISA and ELISPOT, respectively, one week after the last immunization. Fig. 2A demonstrates that CAF01 promoted CMI responses with frequencies of IFN-γ positive cells ranging from 1/4000 to1/5000. This response was primarily directed against the CD4 T cell epitope in OVA (ISQAVHAAHAEINEAGR) (Fig. 2B) whereas the response to the CD8 T cell epitope (SIINFEKL) was at lower levels (Fig. 2C). OVA administered in MPL and DDA also induced significant number of Ag-specific cells but at lower levels compared to CAF01. In MPL-immunized mice this response was primarily seen upon stimulation with the CD8 T cell epitope.

In addition, CAF01 induced OVA-specific IgG1 antibody titers comparable to CFA/IFA, which is the classical immunization regimen for antibody induction in mice, (Fig. 2D) whereas all other adjuvants showed lower levels of humoral responses. In general, the level of IgG2b (Fig. 2E) and IgG2c (Fig. 2F) antibodies was lower compared to IgG1 titers but CAF01 as well as Montanide and CFA/IFA gave rise to detectable levels of these isotypes. Mice receiving OVA alone failed to exhibit an antibody response (results not shown).

### High levels of protection against three diseases with markedly different immunological requirements using CAF01-based vaccines

The ability of CAF01-based vaccines to elicit protection against three different diseases with markedly diverse requirements in terms of balance between CMI and humoral responses was evaluated. Protection against asexual blood-stage malaria is considered to be mediated primarily by antibodies whereas the induction of potent Th1-directed T cell responses is known to be vital in controlling a TB infection. Immunity against chlamydia for comparison relies on the combined action of CMI and humoral responses. For all three diseases, leading antigen vaccine candidates were selected among antigens with a proven track record and used to assess the adjuvant efficacy of CAF01. The antigens were the *Plasmodium yoelii* MSP1-19 antigen (C-terminal 19-kDa processing fragment of MSP1) which is already in clinical trials with different adjuvants [17], [18], the Ag85B-ESAT-6 fusion antigen of *Mycobacterium tuberculosis* which recently went through a phase Ia trial and now has entered a phase Ib trial [19] and the well-established chlamydia vaccine antigen MOMP [20], [21]. A standard immunization scheme was used for all three diseases involving three subcutaneous vaccinations administered with a two-week interval followed by a rest-period between three to six weeks before administration of a virulent challenge administered as outlined in Fig. 1. The immunogenicity was evaluated one week and three weeks after the final vaccination by monitoring the levels of vaccine-specific antibodies in the sera and by evaluating the IFN-γ recall response to the vaccine antigen in splenocyte cultures. For all three antigens the responses were compared to Al(OH)_3_ adjuvanted preparations as this adjuvant is presently the most widely used adjuvant in human vaccines.

**Figure 3:**
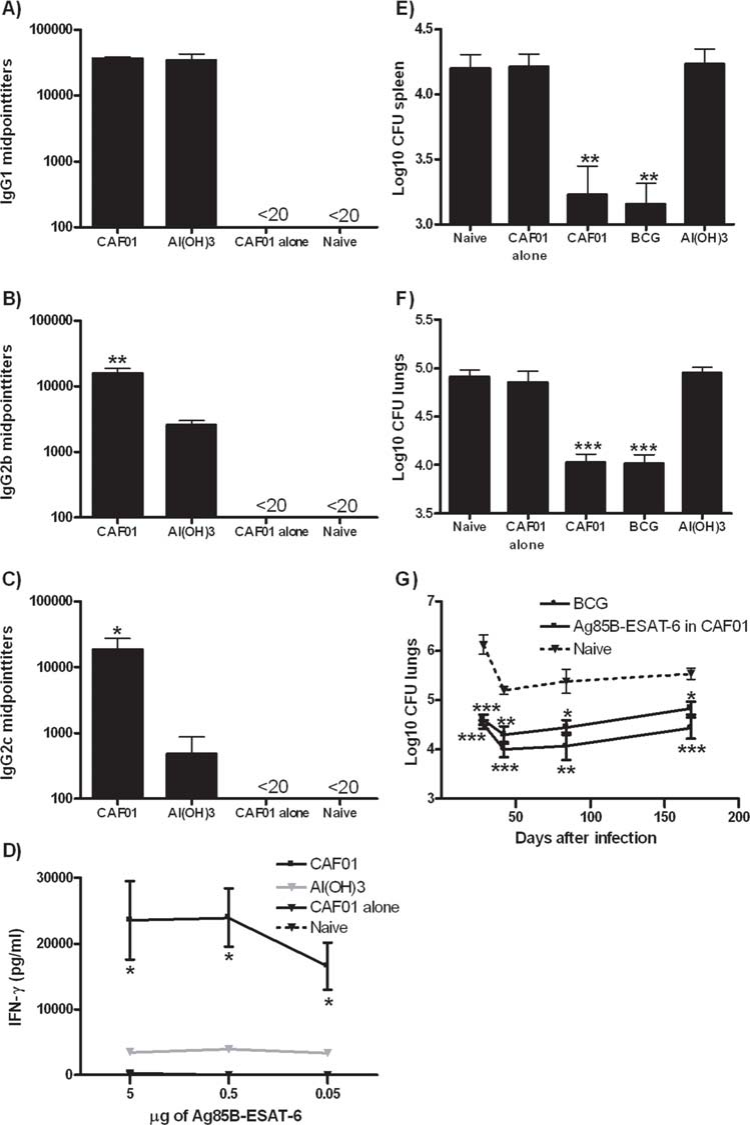
Immune responses and protection induced by CAF01 in a TB model. C57BL/6 mice were immunized three times with 2 µg of Ag85B-ESAT-6 in CAF01 or Al(OH)_3_. A) Three weeks after the last immunization, mice were bled by periorbital puncture and individual sera tested for Ag85B-ESAT-6 IgG1, B) IgG2b or C) IgG2c by ELISA (n = 6). D) Individual cultures of splenocytes (n = 3) were harvested at the same time point and re-stimulated in vitro with different concentrations of the Ag85B-ESAT-6. The release of IFN-γ was determined by ELISA. Six weeks after the last immunization, mice were challenged by the aerosol route with virulent *M. tuberculosis*. Six weeks postchallenge, mice were sacrificed and the bacterial burden (CFU) measured in the E) lungs or F) spleen (expressed as log_10_ CFU). As a positive control group, a group of mice received a BCG vaccination ten weeks before challenge. Data shown are mean values of six mice ± SEM. G) At different time points after infection, mice were sacrificed and the CFU measured in the lungs. Values marked with an asterisk are significantly different (*, *p*<0.05; **, *p*<0.01, *p*<0.001) compared to naïve controls as assessed by ANOVA and Dunnett's multiple comparison test.

*M. tuberculosis*: The mycobacterial fusion protein Ag85B-ESAT-6 in CAF01 induced strong humoral responses with titers of Ag85B-ESAT-6 specific IgG1 antibodies reaching 35,000 which was the same level obtained with the Al(OH)_3_-adjuvanted vaccine (Fig. 3A). The IgG2b (Fig. 3B) and c (Fig. 3C) titers induced by the CAF01 adjuvanted vaccine for comparison was significantly higher than the Al(OH)_3_-promoted responses. No detectable levels of antibodies were seen in mice receiving CAF01 without antigen or in un-vaccinated animals. Ag85B-ESAT-6 in CAF01 was also highly immunogenic when assessing cell-mediated immune responses with levels of more than 20,000 pg/ml IFN-γ released to the supernatant after restimulation of splenocytes with Ag85B-ESAT-6 (Fig. 3D). The IFN-γ response was found to be mediated primarily by CD4+ T cells as restimulation with either the intact Ag85B-ESAT-6 fusion protein or peptides spanning the two proteins followed by intracellular cytokine staining and FACS analysis identified a higher percentage (2–4%) of IFN-γ positive among the CD4+ T cell compared to the CD8+ T cell population (0.3%) (results not shown). Subsequently, the Ag85B-ESAT-6/CAF01, the Ag85B-ESAT-6/Al(OH)_3_ as well as the CAF01 adjuvant control and BCG vaccinated mice were challenged through the aerosol route with *M. tuberculosis* Erdman. Ag85B-ESAT-6 in CAF01 provided significant levels of protection (*p*<0.001 and *p*<0.01 for lungs and spleen, respectively) comparable to that offered by the standard BCG vaccination (Fig. 3E and F). The adjuvant control did not give rise to any non-specific immunity against the TB challenge. As expected, Ag85B-ESAT-6 delivered in Al(OH)_3_ gave rise to low levels of cell-mediated immune responses and failed to provide protection against TB. In order to assess the duration of the CAF01-induced protection, bacterial growth was monitored at different time points (week 4, 6, 12 and 24 after infection) after infection showing the maintenance of approximately one log reduction in bacterial numbers throughout the course of infection at a level almost comparable to that seen in mice receiving the live BCG vaccine (Fig. 3G).

**Figure 4:**
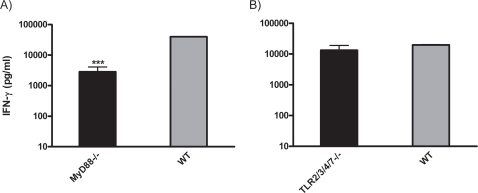
CAF01-induced responses in TLR2, 3, 4, 7 and MyD88-deficient mice. A) MyD88-/- and B) TLR2, 3, 4, 7-/- mice as well as WT controls mice (n = 3) were vaccinated twice with 2 µg of Ag85B-ESAT-6 in CAF01 by footpad immunization. Two weeks after the last immunization, the inguinal lymphnodes were harvested and restimulated in vitro with 10 µg of Ag85B-ESAT-6. The release of IFN-γ was determined by ELISA.

To further study the mechanism of this adjuvant effect of CAF01, we evaluated whether the response was dependent on the MyD88 signalling pathway which is the common TLR-signalling adaptor molecule or dependent on different TLRs including TLR2 and TLR4 known to have a role in the recognition of mycobacterial lipid components. MyD88 deficient mice as well as mice with gene deficiencies in TLR2, 3, 4 and 7 was vaccinated twice with Ag85B-ESAT-6 in CAF01 and the cellular immune response analyzed in the inguinal lymph nodes two weeks after vaccination. As shown in figure 4B, prominent levels of IFN-γ was seen in the TLR2, 3, 4 – and 7 quadruple knock-out mice at a level comparable to what was obtained in wild type animals. In contrast, a significant decrease was observed in MyD88 gene deficient mice (Fig. 4A).

**Figure 5:**
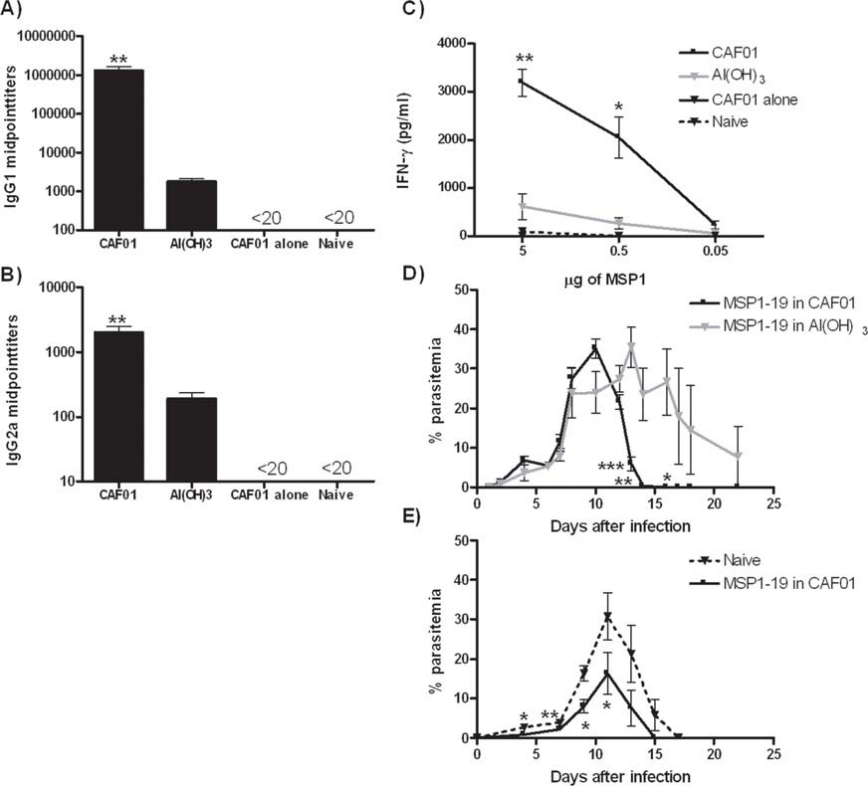
Immune responses and protection induced by CAF01 in a malaria blood-stage model. BALB/c mice were immunized three times with 10 µg of MSP1 in CAF01 or Al(OH)_3_. One week after the last immunization, mice were bled by periorbital puncture and individual sera tested for MSP1-specific A) IgG1 or B) IgG2a by ELISA (n = 5). C) Individual cultures of splenocytes (n = 3) were harvested at the same time point and re-stimulated in vitro with different concentrations of MSP1. The release of IFN-γ was determined by ELISA. D and E) Three weeks after the last immunization, mice were challenged by the i.p. route with *Plasmodium yoelli* and the number of infected red blood cells measured at various time points during infection. Data shown are mean values of five mice ± SEM. Values marked with an asterisk are significantly different (*, *p*<0.05; **, *p*<0.01, *p*<0.001) compared to Al(OH)_3_-vaccinated as assessed by t-test.

*Malaria*: Very high levels of MSP1-19 specific IgG1 and IgG2a antibodies were induced with between 10 (IgG2a) and 700 (IgG1) fold higher levels in the CAF01 adjuvanted MSP1-19 group as compared to the Al(OH)_3_ adjuvanted group (*p*<0.01 for both IgG1 and IgG2a) (Fig. 5A and B). In general, the level of cell-mediated response measured as antigen specific IFN-γ release after *in vitro* re-stimulation with MSP1-19 was lower compared to the Ag85B-ESAT-6 responses obtained but again CAF01 vaccination led to 5–8 times higher response compared to Al(OH)_3_ adjuvanted MSP1-19 (Fig. 5C). No responses were seen in mice receiving MSP1-19 without adjuvant or in un-vaccinated animals. Vaccinated mice received a non-lethal *Plasmodium yoelii* infection through the intraperitoneal route. In the CAF01 group, peak parasitemia occurred around day 10 post challenge with a mean of 35% ± 2.5 after which a clearance of parasites from the circulation was seen with no detectable infected erythrocytes from day 15 and onwards (Fig. 5D). In the Al(OH)_3_ adjuvanted MSP1-19 group a much more protracted course of infection was observed; clearance occurred at a later time point and was not complete even at day 22. In a second experiment, the protection conferred by MSP1-19 in CAF01 was compared to non-vaccinated animals showing significant protection in CAF01-immunized mice from day 4 and onto day 12 (Fig. 5E).

**Figure 6:**
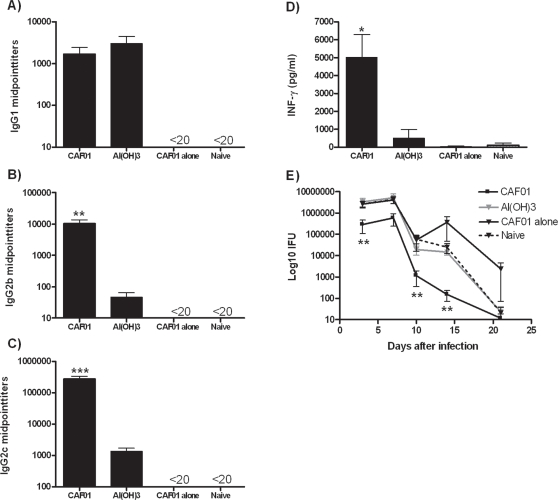
Immune responses and protection induced by CAF01 in a chlamydia model. C57BL/6 mice were immunized three times with 5 µg of MOMP in CAF01 or Al(OH)_3_. Three weeks after the last immunization, mice were bled by periorbital puncture and individual sera tested for MOMP A) IgG1, B) IgG2b and C) IgG2c by ELISA (n = 6). D) Individual cultures of splenocytes (n = 3–6) were harvested at the same time point and re-stimulated in vitro with 5 µg of MOMP. The release of IFN-γ was determined by ELISA. E) Six weeks after the last immunization, mice were administered a vaginal challenge with 1.5×10^5^ *Chlamydia muridarum* IFU/mouse and the bacterial load in the vagina monitored at day 4, 7, 10, 14 and 21. Data shown are mean values of 6–7 mice ± SEM. Values marked with an asterisk are significantly different (*, *p*<0.05; **, *p*<0.01, *p*<0.001) compared to naïve controls as assessed by ANOVA and Dunnetts post test.

*Chlamydia trachomatis:* Upon vaccination with the chlamydia antigen MOMP, the same level of IgG1 titers was seen in the CAF01 and Al(OH)_3_ groups (Fig. 6A). In agreement with the results obtained with the other antigens described above, MOMP in CAF01 led to significantly higher levels of IgG2b and IgG2c (app. 200 fold) compared to MOMP/Al(OH)_3_ (*p*<0.01 and 0.001, respectively) (Fig. 6B and C). Consistent with this antibody isotype distribution, the antigen specific IFN-γ recall response was also significantly elevated in the MOMP/CAF01 group (Fig. 6D). As in the TB model, the CAF01-based vaccine primarily induced a CD4 T cell response (results not shown). No responses were observed in mice receiving CAF01 alone or un-vaccinated control mice. The vaccine-induced protection in MOMP/CAF01 and MOMP/Al(OH)_3_ was assessed by the administration of a vaginal challenge with *Chlamydia muridarum*. The course of infection was followed by enumeration of the number of bacteria in vaginal swabs taken at various time points during infection. Mice that had been vaccinated with MOMP in CAF01 were partially protected and exhibited decreased bacterial load at all time points post infection compared to both un-vaccinated control animals and mice receiving MOMP delivered in Al(OH)_3_ (Fig. 6E). The clearance of bacteria in the Al(OH)_3_ adjuvanted vaccine was identical to that of with naïve controls. In addition, the CAF01-based chlamydia vaccine also resulted in an earlier clearance of bacteria with 2/15 and 5/15 being culture negative already on day 4 and 7 post infection (results not shown). At these time points there was no reduction in the number of culture positive mice in either un-vaccinated control animals or in animals receiving MOMP in Al(OH)_3_.

## Discussion

It is becoming increasingly clear that for peptide- or protein subunit vaccines, amplification of T cell priming through the fine tuned combination of antigen uptake and dendritic cell (DC) activation is necessary to elicit optimal protective immune responses for a large range of diseases. CAF01 is a two-component adjuvant system that consists of an immunomodulator (TDB) and a cationic liposome (DDA) that act in synergy to enhance vaccine-specific immune responses. In this regard, CAF01 share similarities with some of the new promising adjuvant systems that have recently been developed. This includes the GSK series of adjuvants focused on combining immunomodulators like MPL and QS21 with different delivery vehicles e.g. conventional aluminium hydroxide and oil in water emulsions [22]. Others have encapsulated MPL in PLG microparticles [23], CpG in liposomes [24], or ODN1a in cationic peptide based particles [25]. Administered alone the vaccine delivery systems in general induce weak immune responses (as illustrated by DDA alone in Fig. 2) but in combination with an immunomodulator e.g. a TLR agonist a synergistic effect is obtained leading to highly potent and multifaceted immune responses often with both T and B cell responses (reviewed in [26]). In these adjuvant systems, the delivery vesicle serves to promote uptake and presentation of the vaccine antigen in the relevant subset of antigen-presenting cells (APC), whereas the immunomodulator activate the APC through interaction with TLR or non-TLR receptors. This synergy has also been clearly demonstrated for CAF01 where DDA target vaccine antigen to APCs and TDB provides the proinflammatory response for obtaining a Th1 cytokine imprint [27]. Hence, adsorption of OVA onto DDA led to a highly accelerated uptake of antigen by the DCs *in vitro* whereas a neglible uptake was seen with OVA alone [28].

In addition to the synergistic effect on the immune response, another major benefit of co-formulating antigen and immunomodulators into a delivery vehicle is to limit the distribution of the immunostimulatory components and thereby minimize systemic toxicity. This ability of liposomes to target preferentially distinct cells or tissues has been widely used in drug delivery e.g. with Doxil® for cancer therapy [29]. Recently, the same ability was demonstrated for the novel adjuvant system IC31 which is highly effective in promoting cell-mediated immune responses [30]. By following the fate of fluorescently labelled IC31 upon s.c. administration in mice only a minute proportion of the DC population (less than 0.2% of the CD11c+ cells) was found to be taking up the adjuvant. However, these IC31+ DCs were all highly activated with increased levels of CD40, CD80 and CD86 expression. Preliminary observations suggest that CAF01 also elicits a highly targeted and exquisite activation of a minor proportion of the CD11c+ cells (Kamath, manuscript in preparation). In contrast, parenteral administration of soluble CpG leads to a global activation of dendritic cells [30] and highly elevated systemic levels of IL-6, IL-12 and TNF-α with the subsequent risk of serious side effects [31], [32], [33]. Although TDB and its natural analogue trehalosedimycolate (mycobacterial cord factor) has been identified as highly active immunomodulators leading to secretion of IL-1b, IL-6 and TNF-α [34], CAF01 does not have a systemic effect upon injection in mice (levels of IL-6 and TNF-α in sera two hours after injection) and was not pyrogenic in the conventional rabbit pyrogenecity test (results not shown).

TB vaccine development and evaluation has been ongoing for many years and before the detailed knowledge on antigen delivery and DC activation was available, vaccine optimization was performed in an empirical way. It is therefore striking that the three lead adjuvants for TB subunit vaccines AS01 (Mtb72F) [35], IC31 (Ag85B-ESAT-6 and Ag85B-TB10.4) [36], [37] and CAF01 all share the same basic combination of a delivery vehicle and a Th1-inducing immunomodulator. In contrast, single immunomodulators like CpG [38] or MPL [39] as well as delivery vehicles administered without immunomodulators e.g. liposomes or niosomes [40] do not confer significant protection against TB infection.

In chlamydia research, the lack of effective adjuvants has indeed been one of the major impediments for the development of an effective vaccine. Animal challenge experiments showed that parenteral administration of MOMP in either MF59 [41] or aluminium hydroxide (Fig. 6, and [42]) provide limited protection against infection whereas strong CMI responses and efficient protection was promoted by MOMP adjuvanted with a two-component adjuvant (Montanide with CpG) [41].

Whereas the data obtained in both the TB and chlamydia models can be explained by the importance of the CMI responses promoted by CAF01, a similar explanation appears unlikely for blood stage malaria where immunity is most likely exclusively mediated by antibodies [43]. Although the superior protection provided by CAF01 can simply be explained by larger number of antibodies, an additional advantage of CAF01 could be the pronounced Th1-skewed antibody profile with high levels of IgG2 titers. This specific subclass profile is a very consistent observation in all three models and has been associated with the most potent proinflammatory and effective antibody response. In agreement, vaccine-induced IgG2 was previously found particular effective at mediating immunity to blood stage malaria infection in mouse models [44]. Although it is not possible to identify a human analogue, IgG3 shares many characteristics with mouse IgG2 including a more effective anti-malaria response. In epidemiological studies carried out in high endemic areas, the level of IgG3 has been shown to correlate with resistance against the development of clinical malaria [45]. The higher activity of IgG2 has also attracted a lot of interest in other fields including chlamydia where this isotype is found responsible for antibody enhancement of Th1 activation and the subsequent protection [46]. Over the last 5 years, there has been a breakthrough in our understanding of how the various antibody isotypes interact with either activatory or inhibitory Fc receptors and thereby mediate the differential activity observed in vivo [47]. Thus, IgG1 antibodies selectively binds to inhibitory FcγRIIB expressed on dendritic cells whereas IgG2a and IgG2b antibodies preferentially engage the activatory Fcγ:RIV receptor crucial for the higher in vivo activity observed as e.g. enhanced phagocytosis and release of inflammatory mediators [48]. There is therefore a growing interest for the quality of the vaccine-induced antibody response shown to be of crucial importance for the development of the cellular immune response and thereby the protective properties of the vaccine.

Although effective in generating protective B and T-cell responses, a weakness of nonreplicating vaccine adjuvants including CAF01 described herein is the limited CD8 T cell induction against protein antigens. ESAT-6_15–29_ specific CD8 T cell responses can be induced using an adenovirus vector expressing the fusion of Ag85B and ESAT-6 [49], however the CAF01-protein vaccine fails to induce a CD8 T cell response against the same epitope (results not shown). In addition, immunization with OVA/CAF01 led to a strong CD4 T cell response whereas the CD8 T cell response was at lower levels e.g. in comparison to MPL (Fig. 2B and C). However, recently it was shown that by administering type I IFN-inducing TLR ligands e.g. MPL or Poly IC in combination with cationic liposomes a strong CD8 T cell response to protein antigens can be elicited [50]. This has also been demonstrated in human trials where vaccination with a recombinant tumor antigen in CpG/Montanide oil led to CD8 T cell response in a considerable fraction of the patients [51]. The ability of this type of formulation to cross-prime CD8 responses *in vivo* has primarily been attributed to the delivery vehicle mediating efficient interaction between the TLR agonists and their intracellular receptor thereby promoting immune activation [52]. The enrichment of CAF01 with different type I IFN-inducing TLRs is currently under evaluation.

Beyond doubt TLRs have over the last decade become hot targets for vaccine research with the majority of novel adjuvant technologies being based on TLR agonists. To study the role of TLRs in the CAF01-induced immune responses, we vaccinated mice with gene deficiencies in TLR2, 3, 4 and 7 but failed to demonstrate a reduction in vaccine-induced IFN-γ. In contrast, the response in MyD88-/- knock-out mice was reduced indicating that signaling occurs through a different TLR than TLR2, 3, 4, 7. Alternatively, the signaling could occur through a TLR-independent pathway e.g. through the IL1/IL18 receptors known also to utilize the MyD88 signaling pathway or through an yet unidentified receptor upstream of MyD88. Indeed, it has also recently become clear that many classical adjuvants including FCA are capable of inducing an adaptive immune response in the absence of TLR-signalling [53], [54] and therefore CAF01 may be included in this group of adjuvants signaling through TLR-independent pathways.

Our study demonstrates that CAF01 is a very versatile adjuvant system inducing simultaneously a CMI response and an antibody response with high levels of IgG2 antibodies. In the clinical development of liposome-based systems, stability, manufacturing and quality assurance have been major obstacles. However, in addition to the synergy in enhancing immune responses, TDB also has a stabilizing effect on DDA liposomes and the result is a formulation that maintains the same particle size distribution for more than 1½ years at 4°C ([5] and personal communication: Lars Vibe Andreasen). This preparation is currently scheduled to enter clinical trials together with the TB vaccine candidate Ag85B-ESAT6.
